# Compensatory T Cell Responses in IRG-Deficient Mice Prevent Sustained *Chlamydia trachomatis* Infections

**DOI:** 10.1371/journal.ppat.1001346

**Published:** 2011-06-23

**Authors:** Jörn Coers, Dave C. Gondek, Andrew J. Olive, Amy Rohlfing, Gregory A. Taylor, Michael N. Starnbach

**Affiliations:** 1 Department of Molecular Genetics and Microbiology, Duke University, Durham, North Carolina, United States of America; 2 Department of Microbiology and Molecular Genetics, Harvard Medical School, Boston, Massachusetts, United States of America; 3 Departments of Medicine, Molecular Genetics and Microbiology, and Immunology and Center for the Study of Aging, Duke University, Durham, North Carolina, United States of America; 4 Geriatric Research and Education and Clinical Center, Veteran Affairs Medical Center, Durham, North Carolina, United States of America; Yale University School of Medicine, United States of America

## Abstract

The obligate intracellular pathogen *Chlamydia trachomatis* is the most common cause of bacterial sexually transmitted diseases in the United States. In women *C. trachomatis* can establish persistent genital infections that lead to pelvic inflammatory disease and sterility. In contrast to natural infections in humans, experimentally induced infections with *C. trachomatis* in mice are rapidly cleared. The cytokine interferon-γ (IFNγ) plays a critical role in the clearance of *C. trachomatis* infections in mice. Because IFNγ induces an antimicrobial defense system in mice but not in humans that is composed of a large family of Immunity Related GTPases (IRGs), we questioned whether mice deficient in IRG immunity would develop persistent infections with *C. trachomatis* as observed in human patients. We found that IRG-deficient *Irgm1/m3* ^(-/-)^ mice transiently develop high bacterial burden post intrauterine infection, but subsequently clear the infection more efficiently than wildtype mice. We show that the delayed but highly effective clearance of intrauterine *C. trachomatis* infections in *Irgm1/m3* ^(-/-)^ mice is dependent on an exacerbated CD4^+^ T cell response. These findings indicate that the absence of the predominant murine innate effector mechanism restricting *C. trachomatis* growth inside epithelial cells results in a compensatory adaptive immune response, which is at least in part driven by CD4^+^ T cells and prevents the establishment of a persistent infection in mice.

## Author Summary

*Chlamydia trachomatis* is the most common cause of bacterial sexually transmitted disease and can lead to pelvic inflammatory disease, ectopic pregnancy, infertility, and other complications in women. These serious complications have been difficult to study experimentally in laboratory animals because *C. trachomatis* only causes these complications in humans. Previous work in our laboratory has identified two mouse genes responsible for resistance to *C. trachomatis*. Here we consider whether mice lacking the two resistance genes might succumb to the same complications of infection observed in humans. Although initial infection of the mice lacking the two resistance genes is greater in magnitude compared to normal mice, eventually the mice resist serious infection. We found that one component of the mouse immune system, the T cells, expands more robustly to compensate for the lack of the resistance genes.

## Introduction

*Chlamydia trachomatis* is an obligate intracellular bacterial pathogen that causes frequent infections in humans and significant morbidity throughout the world [1]. Ocular infection with *C. trachomatis* is the leading cause of preventable blindness worldwide and genital infection with *C. trachomatis* is the most common bacterial sexually transmitted infection (STI) in the United States [2], [3]. The major complications of *C. trachomatis* genital tract infections arise primarily in women. Acute genitourinary infections with *C. trachomatis* remain asymptomatic in a high proportion of infected individuals, and therefore often go untreated. In a substantial number of infected untreated women *C. trachomatis* can establish persistent infections, which over time result in pelvic inflammatory disease and tubal scarring and can ultimately cause infertility [4], [5].

*C. trachomatis* is a highly specialized, human-adapted pathogen with a narrow host range. Like many other pathogens with a very restricted host range, *C. trachomatis* has evolved to cause persistent infections in its preferred host enabling *C. trachomatis* to establish reservoirs for new infections and assure its survival as a pathogen within the human population [6]. Generally speaking, if a highly specialized pathogen enters a non-typical or “accidental” host, the non-typical host will either succumb to the infection and die or, more commonly, will rapidly clear the infection [7]. However, it is extremely rare for chronic infection to develop in a non-typical, immune-competent host. This basic principle holds true for experimental infections of laboratory mice with *C. trachomatis*. In contrast to human infections, *C. trachomatis* is rapidly cleared from mice when the organisms are instilled in the vagina or directly into the uterus [8], [9]. If it were possible to model elements of human *C. trachomatis* pathogenesis in mice – with a mouse model of chronic human infection - it would accelerate the study of this disease and therapies to combat it. A first step toward this goal is to understand the underlying mechanisms that promote persistent *C. trachomatis* infections in the human host and prevent the establishment of chronic *C. trachomatis* infections in the murine host.

A milestone in dissecting the basis for host tropisms of *C. trachomatis* was the discovery that IFNγ-induced cell-autonomous resistance in epithelial and other non-hematopoietic cell types like fibroblasts fundamentally differs between mice and humans. In human epithelial cells, IFNγ exerts its antimicrobial effect on *C. trachomatis* predominantly through the induction of indole-2,3-dioxygenase (IDO). The enzyme IDO degrades intracellular tryptophan stores, thus starving *C. trachomatis*, a natural auxotroph for tryptophan, of this essential nutrient [10], [11]. In contrast to human cells, most IFNγ-activated murine epithelial cells express insufficient amounts of IDO to restrict bacterial growth, with the notable exception of alveolar epithelial cells [10], [12], [13], [14]. Accordingly, IDO is not required for the clearance of vaginal *C. trachomatis* infections in mice [10], [15]. Instead, mice restrict *Chlamydia* species through a cell-autonomous resistance system that is executed by members of a large family of IFNγ-inducible GTPases called Immunity Related GTPases or IRGs [15], [16], [17], [18], [19], [20]. Remarkably, the divergent IFNγ responses of these two host species, mice and humans, are reflected in the counter-immune mechanisms that exist in two closely related *Chlamydia* species with distinct host tropism. Whereas genital strains of the human pathogen *C. trachomatis* can utilize exogenous indole to produce tryptophan to overcome IDO-mediated growth restriction, the rodent-adapted species *Chlamydia muridarum* has evolved a mechanism to evade IRG-driven immune responses [19], [21]. Given that the human pathogen *C. trachomatis* is highly susceptible to an IRG-driven immune response that is absent in its typical host, humans, but present in epithelial cell and fibroblasts of its non-typical host, mice, we investigated in this study whether the removal of the IRG resistance system would render mice permissive for persistent *C. trachomatis* genital infections.

Here, we report that mice deficient for the expression of two pivotal IRG regulatory proteins, Irgm1 (also called Lrg-47) and Irgm3 (also called Igtp), initially develop high bacterial burden after genital infection compared to wildtype mice. However, in spite of the initial delay in immune clearance, *Irgm1/m3* ^(-/-)^ mice are ultimately able to resolve a *C. trachomatis* infection as rapidly as wildtype mice. An exacerbated CD4^+^ T cell response is essential for the efficient clearance of genital *C. trachomatis* infections in *Irgm1/m3* ^(-/-)^ mice and the prevention of persistence. Our data show that the absence of early innate immune defenses and the resulting unrestricted expansion of *C. trachomatis* trigger an amplified T cell response that results in sterilizing immunity.

## Results

### Deletion of *Irgm3* relieves the expansion defect observed in *Irgm1* ^-/-^ CD4^+^ T cells

**Figure 1:**
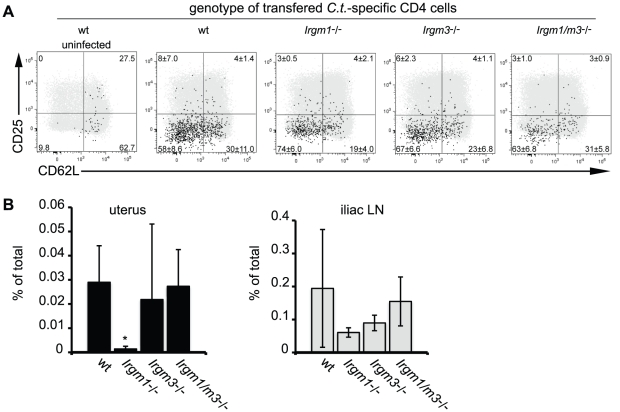
Concomitant deletion of *Irgm3* rescues the expansion defect of *Irgm1* ^-/-^ CD4^+^ T cells. Wildtype B6 mice were injected with naïve, NR1-transgene expressing T cells derived from mice with the indicated genotypes. The next day mice were challenged with 2 * 10^6^ IFUs of *C. trachomatis* directly instilled into the uterus. On day 6 post-infection, flow cytometry was used to analyze cells from the uterus and draining lymph node (A). *C. trachomatis*-specific NR1 cells resident in the iliac lymph nodes were assessed for the expression of activation markers CD25 and CD62L. Total CD4^+^ cells are shown as gray dots, NR1 cells are shown as black dots. (B) The percentage of total live NR1 cells was calculated for uterus (left panel) and draining lymph node (right panel). Flow cytometry data are representative of three independent experiments. Statistical analysis was performed via Student's *t* test. *, *p*<0.05.

**Figure 2:**
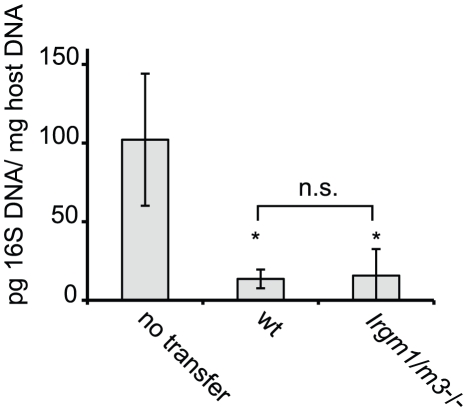
*C. trachomatis*-specific CD4^+^ T cells lacking *Irgm1* and *Irgm3* convey protection to an intrauterine infection with *C. trachomatis.* Th1-skewed wildtype NR1 cells or *Irgm1/m3* ^(-/-)^ NR1 cells were adoptively transferred into mice deficient for IFNγ production (*Ifng* ^-/-^ mice). The following day these mice were transcervically infected with 2 * 10^6^ IFUs of *C. trachomatis*. Three days post-infection, uteri were taken from sacrificed mice and bacterial yield was quantified using qPCR. Data are representative of two independent experiments. Statistical significance relative to the no transfer group is indicated as *, *p*<0.05. n.s.  =  not significant.

We previously reported that *Irgm1* and its paralog *Irgm3* are required for resistance to *C. trachomatis* in an *in vivo* systemic infection model [19]. It is well established that IRG genes like *Irgm1* and *Irgm3* mediate a cell-autonomous antimicrobial response that directly targets vacuolar pathogens like *C. trachomatis* [22], [23], [24]. More recently it has been shown that at least one IRG family member, *Irgm1*, is also required for the proper expansion of CD4^+^ T cells [25]. To determine if *Irgm1* and *Irgm3* deficient mice were more susceptible to *C. trachomatis* infections due to an intrinsic T cell deficiency, we first tested whether expression of Irgm1 and/or Irgm3 in T cells was required for the activation and expansion of CD4^+^ T cells during an intrauterine infection with *C. trachomatis*. Towards this goal we crossed the *C. trachomatis*-specific, MHC class II restricted T cell receptor transgene NR1 onto the *Irgm1* ^-/-^, *Irgm3* ^-/-^ and *Irgm1/m3* ^(-/-)^ genetic backgrounds. From these mice we derived naïve T cells and transferred these T cells into recipient wildtype mice. Although transfer of an antigen experienced or pre-activated population of NR1 cells can accelerate bacterial clearance, the naïve activation state and the low number of *C. trachomatis*-specific T cells transferred in these experiments did not result in accelerated immune clearance, as shown previously [26]. Therefore, the transfer of a relatively small number of IRG-deficient NR1 cells into a wildtype host allowed us to monitor pathogen specific immunity without altering the normal course of the immune response [26], [27]. One day after NR1 cell transfer, we directly instilled *C. trachomatis* into the uterus of these mice by a transcervical infection method. Six days post-infection we monitored the activation and expansion of the transferred NR1 cells in the uterus and the draining lymph nodes of the genital tract. The transferred NR1 cells expressed an allele of the congenic surface marker CD90 that was distinct from the allele expressed by T cells derived from the recipient mice allowing us to specifically detect transferred T cells. We observed that the IRG genotype of the NR1 cells had no apparent impact on the expression of surface activation marker CD62L and CD25 (Fig. 1A) or on the expression of the markers CD127, CD69 and CD44 (data not shown). Similarly, transferred NR1 cells of distinct IRG genotypes were indistinguishable in regards to the proportion of cells expressing the cytokines IFNγ, TNFα and IL-2 (data not shown). However, *Irgm1* ^-/-^ NR1 cells accumulated to significantly lower numbers in the uterus than wildtype NR1 cells did (Fig. 1B), and showed a trend towards lower cell numbers in the draining lymph nodes of the genital tract (Fig. 1B). These data are consistent with the previous observation that *Irgm1*-deficient CD4^+^ T cells fail to expand following an infection in mice [25]. Remarkably, we also found that the expansion defect of *Irgm1* ^-/-^ CD4^+^ cells was reversed by the concomitant removal of *Irgm3* (Fig. 1B), suggesting that *Irgm1/m3* ^(-/-)^ T cells could be fully immune-competent. To directly determine whether *Irgm1/m3* ^(-/-)^ NR1 cells could convey protection to an intrauterine infection with *C. trachomatis,* we transferred Th1 skewed wildtype and *Irgm1/m3* ^(-/-)^ NR1 cells into *Ifng* ^-/-^ mice and subsequently instilled *C. trachomatis* directly into the uterus of these mice. In these experiments the early clearance of the infection is solely dependent on IFNγ secreted by the transferred NR1 cells, since the recipient mice are deficient for IFNγ production [26]. We found that NR1 cells doubly deficient in *Irgm1* and *Irgm3* conveyed protection against intrauterine *C. trachomatis* infection with an efficiency similar to wildtype NR1 cells (Fig. 2). In sum, these data indicated that the removal of *Irgm3* salvaged the T cell intrinsic defect of *Irgm1* ^-/-^ cells and that *Irgm1*/*3* ^(-/-)^ CD4^+^ T cells were fully functional.

**Figure 3:**
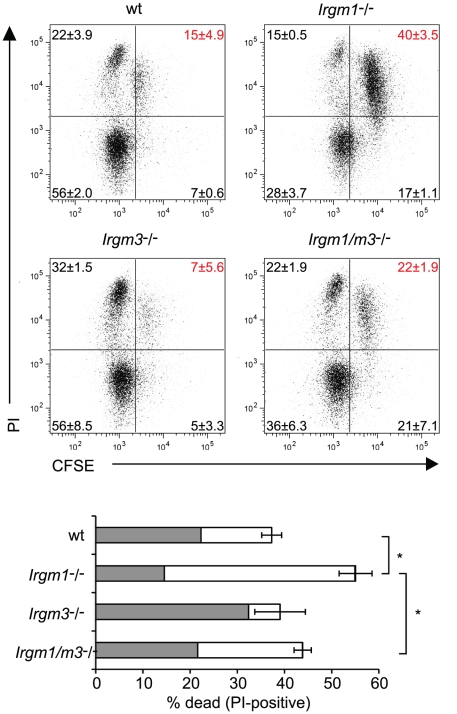
Concomitant deletion of *Irgm3* rescues the cell death phenotype of *Irgm1* ^-/-^ CD4^+^ T cells. Flow cytometry of CFSE-labeled wildtype, *Irgm1* ^-/-^, *Irgm3* ^-/-^ and *Irgm1/m3* ^(-/-)^ CD4^+^ T cells that had been activated with anti-CD3 and anti-CD28 antibodies in the presence of IL-2 for 72 hours and were subsequently stained for dead cells with propidium iodide. Percentages and standard deviations are given for each quadrant (upper panel). The total percentage of propidium iodide positive cells is shown for each genotype. The filled area of the bar indicates the fraction of CFSE^low^ cells and the open area marks the fraction of CFSE^high^ cells (lower panel). Data are representative of three independent experiments. Statistical significance for the percentages of total cell death is indicated as *, *p*<0.05.

The inability of *Irgm1* ^-/-^ NR1 cells to expand to similar numbers as wildtype NR1 cells at the site of the infection could be explained by the propensity of *Irgm1*-deficient mature CD4^+^ T cells to prematurely die when activated for proliferation [25]. Considering that the concomitant removal of *Irgm3* ‘rescues’ the T cell expansion defect of *Irgm1* ^-/-^ NR1 cells, we hypothesized that the simultaneous deletion of *Irgm3* might serve to reverse the premature cell death phenotype of *Irgm1* ^-/-^ CD4^+^ T cells. To test this idea, we labeled wildtype, *Irgm1* ^-/-^, *Irgm3* ^-/-^ and *Irgm1/m3* ^(-/-)^ CD4^+^ T cells with CFSE and then activated them for proliferation using plate-bound anti-CD3 and anti-CD28 antibodies for 72 hours. As reported previously [25], we observed increased total cell death of *Irgm1* ^-/-^ T cells compared to wildtype cells using propidium iodide incorporation as a measure for cell death (Fig. 3). The increase in cell death in *Irgm1* ^-/-^ T cells compared to wildtype cells was most pronounced in the population of CFSE^high^ cells that had only undergone a few rounds of cell division. However, *Irgm1/m3* ^(-/-)^ T cells showed a significant decrease in cell death compared *Irgm1* ^-/-^ T cells and the total percentage of dead *Irgm3* ^-/-^ and *Irgm1/m3* ^(-/-)^ T cells was similar to wildtype T cells (Fig. 3). Taken together, these data showed that the deletion of *Irgm3* reverses the T cell expansion and cell survival defect found in *Irgm1*-deficient T cells.

### *Irgm1/m3* ^(-/-)^ fibroblasts are deficient for IFNγ-induced cell-autonomous resistance to *C. trachomatis*

**Figure 4:**
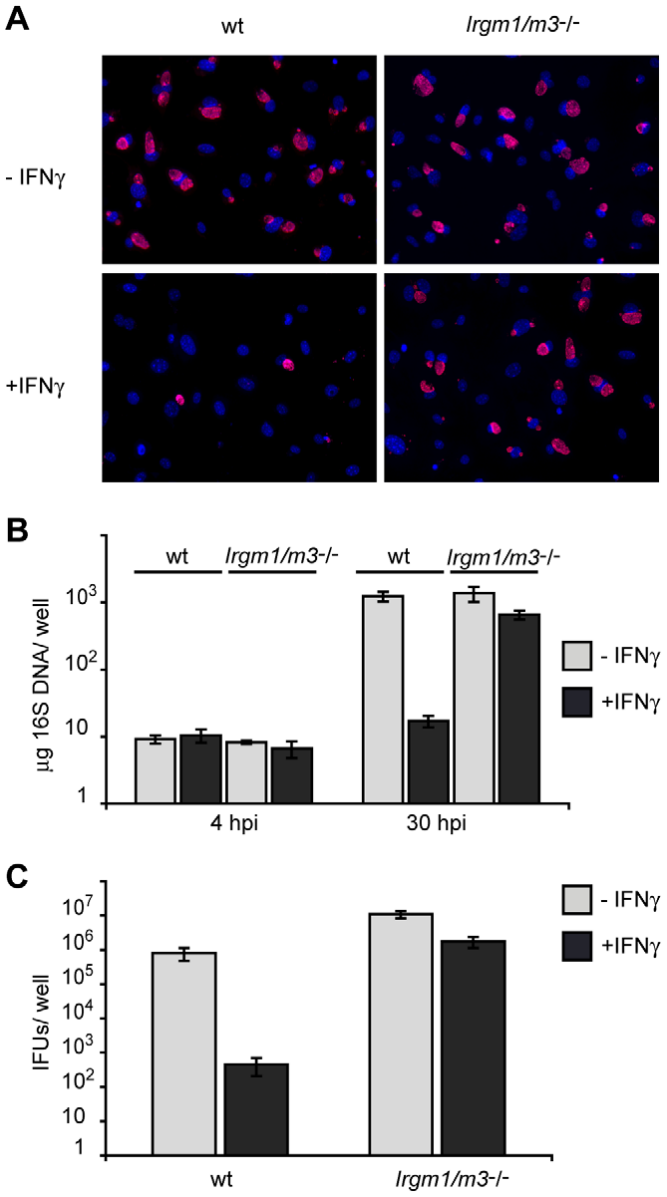
Cells doubly deficient for *Irgm1* and *Irgm3* exert minimal IFNγ-induced cell-autonomous resistance to *C. trachomatis*. IFNγ-stimulated and unstimulated MEFs derived from wildtype B6 or *Irgm1/m3* ^(-/-)^ mice were infected with *C. trachomatis* at an MOI of 2. (A) Cell nuclei were stained with DAPI and *C. trachomatis* inclusions were labeled using an anti-Hsp70 antibody (B) Bacterial growth was quantified using qPCR at 4 and 30 hpi and (C) using an inclusion-forming unit (IFU) assay at 48 hpi. Data is representative of at least two independent experiments.

IFNγ-activated cells lacking Irgm1 expression are impaired in their ability to contain the intracellular growth of various intracellular pathogens including *Salmonella typhimurium*, *Toxoplasma gondii* and *C. trachomatis* [22], [23], [24]. Recently, it has been shown that *Irgm1*-deficient cells can regain the ability to fully restrict growth of *S. typhimurium* when an additional genetic lesion in *Irgm3* is introduced, suggesting that *Irgm1* and *Irgm3* are not essential to restrict *S. typhimurium* inside an infected cell [28]. In contrast, IFNγ-activated cells doubly deficient in *Irgm1* and *Irgm3* remain at least as susceptible to a *T. gondii* infection as cells harboring single *Irgm* gene deletions [28]. To determine whether *Irgm1* and *Irgm3* are essential for IFNγ-induced cell-autonomous resistance to *C. trachomatis*, we generated *Irgm1/m3* ^(-/-)^ mouse embryonic fibroblasts (MEFs). MEFs lacking both *Irgm1* and *Irgm3* displayed a near complete loss of IFNγ-induced resistance to *C. trachomatis* growth. Whereas only very few *C. trachomatis* inclusion were detectable in IFNγ-activated wildtype MEFs, the number of inclusions found in IFNγ-treated and untreated *Irgm1/m3* ^(-/-)^ MEFs was similar (Fig. 4A). To more accurately quantify *C. trachomatis* replication in these MEFs, we harvested DNA from infected cells at 4 and 30 hours post-infection (hpi) and measured the amount of *Chlamydia* DNA using qPCR. Neither IFNγ treatment nor IRG deficiency had any appreciable effect on *C. trachomatis* burden at 4 hpi, suggesting normal bacterial attachment and entry under all conditions. By 30 hpi IFNγ activation, *C. trachomatis* yields were reduced by nearly 2 logs in wildtype MEFs. In contrast, we only observed a 2-fold reduction in IFNγ-treated *Irgm1/m3* ^(-/-)^ MEFs (Fig. 4B). Because it was formally possible that *C. trachomatis* was able to replicate but unable to differentiate into infectious elementary bodies in IFNγ-activated *Irgm1/m3* ^(-/-)^ MEFs, we also assayed the release of infectious EBs by monitoring the production of inclusion forming units (IFUs) in these cells. Consistent with our findings by qPCR (Fig. 4B), we found that the ability of IFNγ-activated MEFs to restrict the production of infectious *C. trachomatis* progeny was severely compromised in *Irgm1/m3* ^(-/-)^ MEFs (Fig. 4C). Collectively, our results showed that *Irgm1/m3* ^(-/-)^ MEFs have lost their ability to efficiently restrict growth of *C. trachomatis* upon IFNγ activation.

Because these experiments were conducted in tryptophan-rich culture media, we could not dismiss a possible role for the tryptophan-degrading enzyme IDO in restricting *C. trachomatis* growth in IFNγ-activated MEFs. To test for such a function of IDO, we first measured induction of mouse IDO1 expression upon IFNγ activation. In contrast to the approximately 1000-fold induction of Irgm3 and Irgb10 mRNA expression, IDO1 mRNA was only moderately induced (5-fold), and more importantly, inhibition of *C. trachomatis* growth upon IFNγ treatment remained unchanged in the presence or absence of tryptophan in the culture media (Fig. S1A and B). Because ectopic expression of IDO resulted in significant restriction of *C. trachomatis* growth in MEFs (Fig. S1C), we conclude that endogenous IDO expression levels are too low in IFNγ-stimulated MEFs to restrict *C. trachomatis* growth.

### Initially delayed but subsequently accelerated clearance of intrauterine *C. trachomatis* infections in *Irgm1/m3* ^(-/-)^ mice

**Figure 5:**
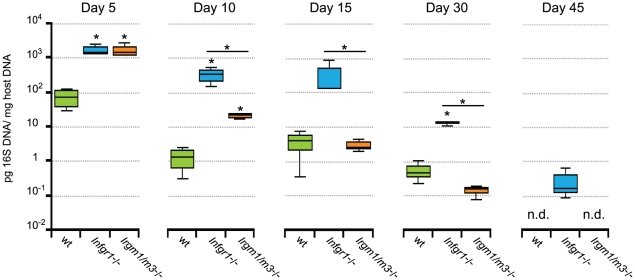
*Irgm1/m3* ^(-/-)^ mice show delayed but efficient clearance of genital *C. trachomatis* infections. B6 wildtype mice and the coisogenic knockout strains B6.*Ifngr1* ^-/-^, and B6.*Irgm1/m3* ^(-/-)^ were transcervically infected with 2 * 10^6^ IFUs of *C. trachomatis*. At the indicated days post-infection 3 to 4 mice of each genotype were sacrificed and bacterial burden in the uteri was quantified by qPCR. Data were combined from two independent experiments. Statistical analysis of box-and-whisker plots was done via Student's *t* test and significant difference relative to wildtype mice and between *Ifngr1* ^-/-^, and *Irgm1/m3* ^(-/-)^ are indicated by *, p<0.05. The limit of detection was approximately 10^−4^ pg 16S DNA/µg host DNA.

Since MEFs isolated from *Irgm1*/*3* ^-/-^ mice failed to constrain *C. trachomatis* growth in the presence of IFNγ, we expected that these mice would also be unable to control *C. trachomatis* genital infections. To test this idea, we infected wild type, *Ifngr1^-/-^*, and *Irgm1/m3* ^(-/-)^ mice transcervically with *C. trachomatis* and measured the bacterial burden in the genital tract over 45 days. As expected, mice lacking the gene encoding the IFNγ receptor (*Ifngr1^-/-^*) showed greater bacterial burden than wildtype mice over the entire time course (Fig. 5). Initially *Irgm1/m3* ^(-/-)^ mice also developed high bacterial burden similar to the burden observed in *Ifngr1^-/-^* mice, yet by day 15 post-infection the number of organisms present in *Irgm1/m3* ^(-/-)^ mice was reduced to levels similar to the ones found in wildtype mice (Fig.5). By day 45 post-infection, *C. trachomatis* was still detectable in *Ifngr1^-/-^* mice but undetectable in wildtype and *Irgm1/m3* ^(-/-)^ mice. These data show that although *Irgm1/m3* ^(-/-)^ mice were initially defective in clearing *C. trachomatis* infections, they ultimately were able to control the infection. Therefore, we conclude that *Irgm1/m3* ^(-/-)^ mice do not exhibit an infection of extended duration as had been observed with the *Ifngr1^-/-^* mice.

### An exacerbated CD4^+^ T cell response mediates the clearance of intrauterine *C. trachomatis* infection in *Irgm1/m3* ^(-/-)^ mice

**Figure 6:**
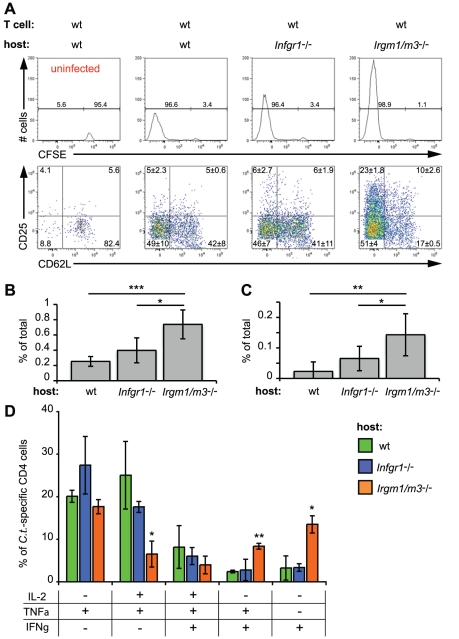
The absence of *Irgm1/3*-dependent immunity results in an exacerbated CD4^+^ T cell response. CFSE-labeled, wildtype NR1cells were adoptively transferred into recipient mice of the indicated genotypes. The mice were subsequently transcervically infected with 2 * 10^6^ IFUs of *C. trachomatis*. On day 6 post-infection, flow cytometry was used to analyze cells from the uterus and draining lymph node. Representative data of four independent experiments are shown. (A) A total of 10^6^ lymphocytes were collected in each sample and the absolute number of CD4^+^ CD90.1^+^ NR1 cells was analyzed for CFSE dye dilution (upper panel) and expression of the surface proteins CD25 and CD62L (lower panel). All cells derived from the draining lymph nodes were used for the analysis. The number of *C. trachomatis*-specific NR1 cells is shown as the percentage of total number of cells in the iliac lymph node (B) and the uterus (C). (D) NR1 cells harvested from the iliac lymph node were also restimulated for 5 h with PMA-ionomycin and assessed for intracellular cytokine staining. Statistical significance relative to wildtype recipient mice is shown. *, p<0.05; **, p<0.01; and ***, p<0.005.

We then began to investigate how *Irgm1/m3* ^(-/-)^ mice were able to clear *C. trachomatis* infections despite the initially high bacterial burden observed at day 5 and earlier. One possible explanation was that the increased antigen burden in the *Irgm1/m3* ^(-/-)^ mice stimulates the expansion and activation of *C. trachomatis*-specific effector T cells. To test the idea that host *Irgm1*/*m3*-deficiency could impact the development of wildtype T cells, we transferred naïve, CFSE-labeled, wildtype *C. trachomatis*-specific NR1 cells into wildtype, *Ifngr1^-/-^* or *Irgm1/m3* ^(-/-)^ host mice. Although we had already shown that *Irgm1/m3* ^(-/-)^ T cells appear to be indistinguishable from wildtype T cells (Fig. 1 and 2), we opted to use wildtype NR1 cells in these experiments in order to exclude any cell-autonomous effects on T cells that could be caused by the presence of the *Ifngr1^-/-^* or the *Irgm1/m3* ^(-/-)^ alleles. One day after the transfer of wildtype NR1 cells into mice of the indicated genotypes, mice were infected with *C. trachomatis* in the uterus. We found that *C. trachomatis* infection stimulated robust proliferation of transferred NR1 cells regardless of the genotype of the recipient mice (Fig. 6A, top panel). Though the genotype of the recipient mouse had no discernable impact on NR1 cell proliferation based on CSFE dye dilution, we did observe a significant increase in the total number of NR1 cells in the iliac lymph node (Fig. 6A and 6B) and the uterus (Fig. 6C) of *Irgm1/m3* ^(-/-)^ mice on day 6 post-infection compared to *Ifngr1* ^-/-^ and wildtype mice. The greater expansion of NR1 cells in *Irgm1/m3* ^(-/-)^ mice corresponded to an increase in the population of NR1 cells expressing the IL-2 high affinity receptor CD25 on NR1 cells resident in *C. trachomatis*-infected *Irgm1/m3* ^(-/-)^ mice compared to infected *Ifngr1* ^-/-^ and wildtype mice (Fig. 6A). Additionally, a significantly larger proportion of NR1 cells expressed IFNγ, the hallmark cytokine of Th1 activation, and the proinflammatory cytokine TNFα in combination with IFNγ, when transferred into *Irgm1/m3* ^(-/-)^ recipient mice compared to *Ifngr1* ^-/-^ and wildtype recipient mice (Fig. 6D). As expected for conventional Th1 cells, we did not detect significant IL-17 expression in NR1 cells transferred into either wildtype or *Irgm1/m3* ^(-/-)^ mice on day 6 post-infection (data not shown).

**Figure 7:**
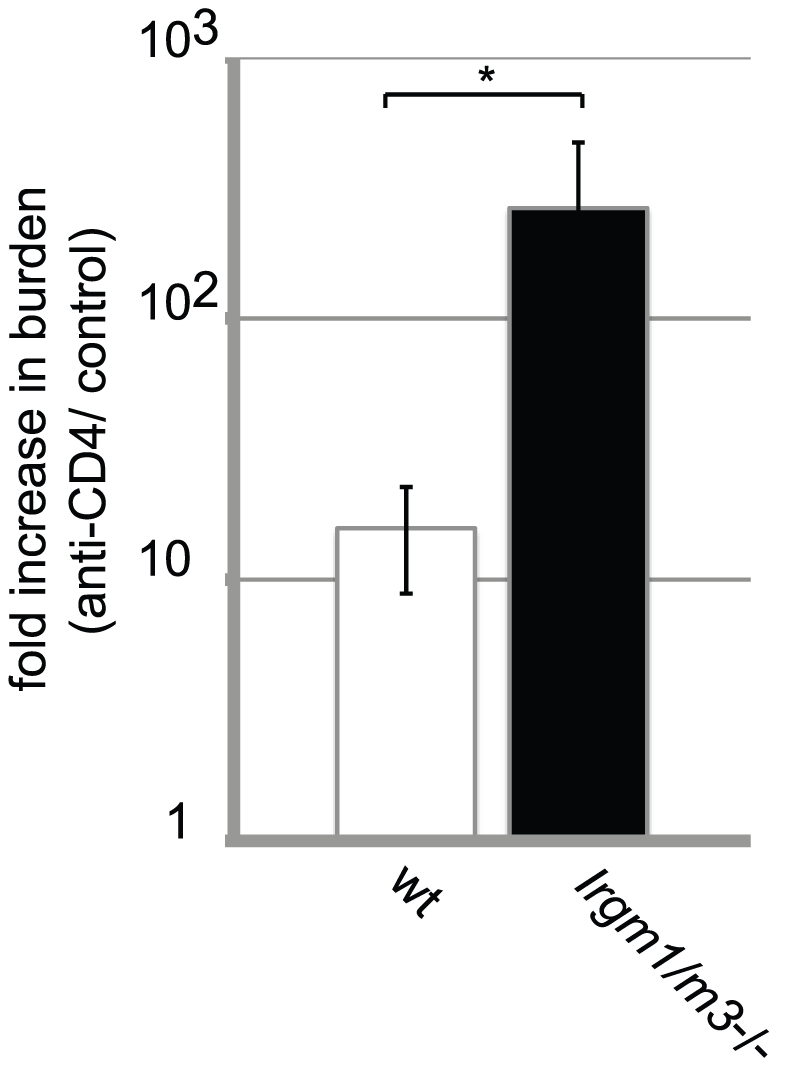
Dependency on the CD4^+^ T cell response for clearing intrauterine *C. trachomatis* infections is increased in *Irgm1/m3* ^(-/-)^ mice. Groups of wildtype B6 and *Irgm1/m3* ^(-/-)^ mice were treated with either ant-CD4 depleting or control antibodies as described in Materials and Methods. At 15 days post-infection mice were sacrificed and bacterial burden in the uteri was determined by qPCR. The fold increase in bacterial burden following treatment with anti-CD4 antibody compared to control antibody-treated mice is shown. The increase in burden in anti-CD4-treated *Irgm1/m3* ^(-/-)^ mice was significantly higher than in wildtype mice (*, p<0.05). Data are representative of three independent experiments.

These results suggested that enhanced expansion of *C. trachomatis*-specific T cells and a boost in their activation status may compensate for the loss of innate immune restriction due to the *Irgm1* and *Irgm3* mutations. To prove that a compensatory amplification of the T cell response was responsible for clearance of *C. trachomatis* from the genital tract, we subjected groups of *Irgm1/m3* ^(-/-)^ and wildtype mice to multiple treatments with either anti-CD4 depleting or control antibodies and then determined bacterial burden in the uterus at 15 days post-infection. As expected, depleting CD4^+^ T cells resulted in greater bacterial burden in both wildtype as well as *Irgm1/m3* ^(-/-)^ mice relative to control mice of the same genotype, confirming that the adaptive immune response contributes to immune clearance at later times in the course of the infection (Fig. 7). Depletion of CD4^+^ T cells, however, had a greater effect on bacterial burden in *Irgm1/m3* ^(-/-)^ mice than it did in wildtype mice, increasing bacterial yield in the uterus by two logs instead of one log. These data confirmed that *Irgm1/m3* ^(-/-)^ mice compared to wildtype mice developed an exacerbated T cell response that was instrumental in the rapid clearance of genital infection with *C. trachomatis*.

### Neutrophil infiltration to the uterus is enhanced in *C. trachomatis* infected *Irgm1/m3* ^(-/-)^ mice compared to wildtype mice

We then began to investigate the mechanism by which an exacerbated CD4^+^ T cell response clears *C. trachomatis* infections in mice lacking cell-autonomous IRG resistance in epithelial cells. Because of the recent demonstration that *Chlamydia*-specific T cells can control *Chlamydia* replication inside epithelial cells by a Nos2-dependent mechanism [29], we tested whether Nos2 was responsible for the elimination of *C. trachomatis* in *Irgm1/m3* ^(-/-)^ mice. To test this hypothesis, we generated *Irgm1/m3* ^(-/-)^ *Nos2^(-/-)^* triple knockout mice. Although *C. trachomatis*-infected *Irgm1/m3* ^(-/-)^ *Nos2^-/-^* mice trended towards higher bacterial burden compared to *Irgm1/m3* ^(-/-)^ or wildtype mice, this effect was not statistically significant in our experiments (Fig. S2).

**Figure 8:**
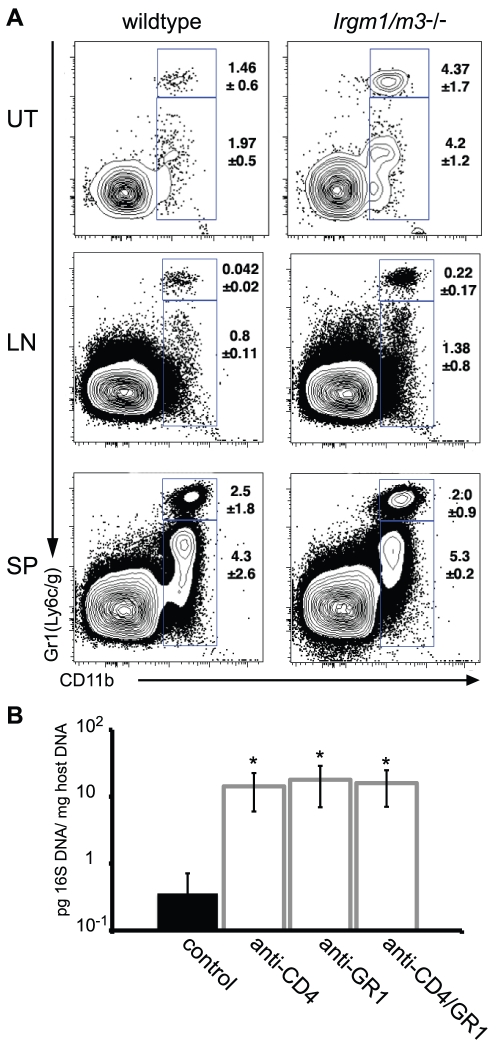
A neutrophil surge into the uterus of *C. trachomatis*-infected *Irgm1/m3* ^(-/-)^ mice plays a role in immune clearance. (**A**) Wildtype or *Irgm1/m3* ^(-/-)^ mice were transcervically infected with *C. trachomatis*. Fifteen days later, the uterus (UT), iliac lymphnodes (LN), and spleens (SP) of these mice were assessed for lymphocyte populations via FACS analysis. Macrophage populations were gated via CD11b^+^GR1^low^, while neutrophil populations exhibited a CD11b^+^GR1^high^ phenotype. Average percent of total is represented on the right of the gated population +/- standard deviation. Neutrophil populations in uteri and lymph nodes were statistically different between wildtype and *Irgm1/m3* ^(-/-)^ mice (p<0.05). (B) *Irgm1/m3* ^(-/-)^ mice were treated with depleting antibodies as described in Materials and Methods and 15 days post-infection bacterial burden in the spleen was measured by qPCR. Significant increases in bacterial burden in antibody-treated *Irgm1/m3* ^(-/-)^ mice compared to mice treated with an isotype control antibody are indicated (*, p<0.05). The data are representative of two independent experiments.

While exploring alternative CD4-dependent immune mechanisms targeting *C. trachomatis* in *Irgm1/m3* ^(-/-)^ mice, we identified an increase in the uterine population of cells expressing the neutrophil surface marker GR1 relative to wildtype mice at day15 post-infection. An increase in the number of GR1^+^ cells was also observed in the iliac lymph nodes but not in the spleen of *Irgm1/m3* ^(-/-)^ mice (Fig. 8A). Similar to the depletion of CD4^+^ T cells, the depletion of the GR1^+^ cell population or the simultaneous depletion of CD4^+^ and GR1^+^ cells in *Irgm1/m3* ^(-/-)^ mice resulted in a dramatic increase in bacterial burden (Fig. 8B), suggesting an important role for neutrophils in the elimination of *C. trachomatis* in *Irgm1/m3* ^(-/-)^ mice.

Lastly, we explored the question as to why *Irgm1/m3* ^(-/-)^ mice cleared *C. trachomatis* infections more efficiently than *Ifngr1* ^-/-^ mice did (Fig. 5). Both *Irgm1/m3* ^(-/-)^ and *Ifngr1* ^-/-^ mice lack IRG-mediated cell-autonomous resistance, yet differ in the number and activation status of their *Chlamydia*-specific T cell population (Fig. 6). To determine whether T cells in conjunction with neutrophils are responsible for the relatively more efficient elimination of *C. trachomatis* in *Irgm1/m3* ^(-/-)^ mice compared to *Ifngr1* ^-/-^ mice, we treated *Irgm1/m3* ^(-/-)^, *Ifngr1* ^-/-^ and wildtype mice with anti-CD4 and anti-GR1 depleting antibodies, and determined bacterial burden at 15 days post infection. As expected, bacterial burden was dramatically elevated in all CD4/GR1-depeleted animals regardless of their genetic background. However, the most dramatic effect on bacterial burden was observed in *Irgm1/m3* ^(-/-)^ mice (Fig. S3) elevating the bacterial yield to the same levels observed in similarly treated *Ifngr1* ^-/-^ mice. These results suggest that IFNγ directs T cell- and neutrophil-dependent clearance of *C. trachomatis* infections.

## Discussion

Genital infections with *C. trachomatis* are among the most common STI worldwide and constitute the most frequent bacterial STI in the United States. Asymptomatic and consequently unrecognized and untreated *C. trachomatis* infections can ascend from the cervix to the fallopian tube and establish persistence resulting in irreversible tissue damage and infertility [30], [31]. In contrast to humans, mice clear genitourinary infection with *C. trachomatis* rapidly and members of the IRG gene family play an important role in conveying resistance to *C. trachomatis* infections in the mouse. Whereas the importance for IRG proteins in resistance to *C. trachomatis* infections in mice is undisputed, the function of the constitutively expressed human ortholog IRGM in the pathogenesis of human *C. trachomatis* infections is less clear. Although IRGM induces antimicrobial xenophagy in human cells [32], [33], [34], [35], most human cells restrict growth of *C. trachomatis* by an IDO-dependent and apparently IRGM-independent mechanism ([10], [11], [15] and Fig. S1B). Although IRGM may still prove to be important in providing resistance to *C. trachomatis* infections in some cell types or tissues, IRGM is less likely to play a prominent role in the pathogenesis of human *C. trachomatis* infections. For these reasons, we sought to determine whether mice deficient in IRG-dependent resistance to *C. trachomatis* - and thus resembling humans in that regard - would develop persistent *C. trachomatis* infections.

We have previously shown that two members of the IRG gene family, *Irgm1* and *Irgm3*, mediate resistance to *C. trachomatis* in an *in vivo* model of systemic infection [19]. Because the gene *Irgm1* not only conveys cell-autonomous resistance to vacuolar pathogens inside an infected host cell but is also required to prevent the premature cell death of activated, proliferating CD4^+^ T cells, we first tested the hypothesis that the inability of IRG-deficient mice to efficiently restrict *C. trachomatis* infections could partly be due to a diminished CD4^+^ T cell response. We found that *Irgm1* ^-/-^ CD4^+^ T cells specific for a *C. trachomatis* epitope failed to efficiently expand in the uterus of genitally infected animals and prematurely died when activated for proliferation *ex vivo.* These results are consistent with the previously described expansion defect of *Irgm1* ^-/-^ CD4^+^ T cells [25]. However, we also found that the simultaneous removal of *Irgm3* in *Irgm1/m3* ^(-/-)^ CD4^+^ T cells ‘rescued’ the phenotype of *Irgm1* ^-/-^ CD4^+^ T cells. Two alternative models may account for these observations: the first model is based on the published finding that IFNγ-induced IRG proteins form large protein aggregates in the absence of Irgm1 expression [36], [37]. These protein aggregates are not found in IFNγ-activated wildtype cells and are likely to have cytotoxic properties. The concomitant removal of *Irgm3* has been shown to reduce the levels of IRG protein aggregates inside a cell, possibly below a threshold level of toxicity [28]. Because T cells have a relatively small cytoplasmic volume compared to most differentiated cells, they may be particularly susceptible to the cytotoxic effects of IRG protein aggregates. According to the “aggregate model” recently proposed by Hunn and Howard [38], the expansion defect of *Irgm1* ^-/-^ CD4^+^ T cells could be due to the cytotoxicity of IRG protein aggregates that form when the finely balanced network of IRG protein interactions is artificially disrupted by the *Irgm1* gene deletion. In a second, alternative, model IFNγ-induced IRG mediated cell death in T cells is a regulated biological process that plays an important role in fine-tuning T cell homeostasis. Our observations can be reconciled with a model in which Irgm3 protein acts as an inducer of cell death whereas Irgm1promotes cell survival, functioning as an Irgm3 antagonist. According to this model, regulated changes in the expression of Irgm1 relative to Irgm3 or post-translational modifications of either Irgm protein could shift the balance towards either T cell survival or T cell death in wildtype T cells. Removal of the pro-survival factor Irgm1 in *Irgm1* ^-/-^ T cells would result in uncontrolled cell death due to the unrestricted action of Irgm3. Further removal of the pro-death factor Irgm3 in *Irgm1*/*3* T cells would restore cell survival. In support of the latter model, we found that anti-CD3 stimulated *Irgm3* ^-/-^ CD4^+^ T cells showed improved cell viability compared to wildtype T cells at least during the first few rounds of anti-CD3 stimulated cell division (Fig. 2), suggesting that Irgm3 may indeed function as a pro-cell death molecule. Additionally, we observed that Irgm3^-/-^ mice (but not *Irgm1* ^-/-^ or wildtype mice) expressing the NR1 transgene relatively frequently developed lymphoma with advanced age (JC and MNS, unpublished data), indicating a potential role for *Irgm3* as a tumor suppressor gene. Beyond the uncertainty of the biological functional importance of IRG proteins in murine T cells, the additional unanswered question remains as to whether IRGM, the single human IRG ortholog constitutively and ubiquitously expressed in human cells, plays any role in regulating T cell homeostasis.

Because *Irgm1*/*3* double deficiency does not intrinsically affect T cell expansion or function, we were able to investigate whether the absence of IRG-mediated resistance in epithelial cells in an otherwise immune-competent host would impact the course and duration of a genital *C. trachomatis* infection. We found that *Irgm1/m3* ^(-/-)^ mice initially failed to effectively clear *C. trachomatis* during the early course of an infection. This observation can be explained with a nearly complete defect in IFNγ-induced cell-autonomous resistance in cells doubly deficient in *Irgm1* and *Irgm3* (Fig. 3). At later time points, however, *Irgm1/m3* ^(-/-)^ mice cleared genital *C. trachomatis* infections rapidly due to an exacerbated *C. trachomatis*-specific CD4^+^ T cell response. The observation that *Irgm1/m3* ^(-/-)^ mice developed a more pronounced T cell response towards *C. trachomatis* than *Ifngr1* ^-/-^ mice did, can be explained by the important role IFNγ plays in stimulating the adaptive immune response, for example, through improved antigen presentation allowing for direct cytolysis by degranulating CD4 T cells [29] and/or enhanced neutrophil recruitment and survival [39], [40], [41], [42]. It has also been reported that IFNγ acts as a positive regulator of CD25 expression on CD4+ T cells in a mouse model of myocarditis, though it is unclear whether IFNγ directly or indirectly controls CD25 expression in CD4+ T cells [43]. The boost in CD25 surface expression on wildtype NR1 cells resident in *C. trachomatis*-infected *Irgm1/m3* ^(-/-)^ mice compared to *C. trachomatis*-infected *Ifngr1* ^-/-^ mice may therefore be the net result of high antigen burden and simultaneous IFNγ activation of antigen presenting cells. In addition to an increase in CD25 expression on NR1 cells, we also observed greater accumulation of NR1 cells in *C. trachomatis* infected *Irgm1/m3* ^(-/-)^ mice compared to *C. trachomatis* infected wildtype or *Ifngr1* ^-/-^ mice. It seems likely that these two phenotypes are linked: the surface protein CD25 is identical to the alpha chain of the high-affinity, trimeric IL-2 receptor and expression of CD25 has been shown to be upregulated on activated conventional T cells [44], [45]. Although signaling through the IL-2 receptor is not essential for T cell effector function *in vivo*, IL-2 is known to promote T cell survival and expansion [46], [47], [48], [49]. The increase in CD25 expression in NR1 cells and the resulting boost in IL-2 mediated cell survival may therefore be the underlying cause for the greater expansion of NR1 cells that we observed in *C. trachomatis* infected *Irgm1/m3* ^(-/-)^ mice. Collectively, these data indicate that the virtual absence of cell-autonomous host resistance during the early course of a *C. trachomatis* infection triggers a compensatory adaptive immune response in an otherwise fully immune-competent host and ultimately prevents the establishment of a sustained genital infection with *C. trachomatis* in mice.

These results prompt the question of how *C. trachomatis* can prevent clearance by the adaptive immune response in its natural host, humans, which lack a robust IRG-dependent immune response that targets *C. trachomatis* directly. We propose that the answer to this question lies in the adaption of *C. trachomatis* to a different cell-autonomous IFNγ response – one found in humans where the pathogen has evolved but absent from urogenital epithelial cells in mice [50]. A wealth of *in vitro* experimental data shows that IFNγ activation of human epithelial cells results in the expression of IDO, which leads to the depletion of intracellular tryptophan stores [10], [51], [52], [53], [54]. In response to tryptophan starvation, *C. trachomatis* transforms from an active, replicating, state into a quiescent form [55]. Replication of these organisms stops, yet they endure in a quiescent form until immune response wanes and tryptophan becomes available again [4], [5], [55], [56], [57]. The IDO-induced, non-replicating quiescent organisms are less likely to trigger a strong adaptive immune response due to reduced antigenic burden. Additionally, the quiescent bacterium may be more resistant to immune effector mechanisms than its replicating form. We therefore propose that in order to avoid clearance by the adaptive immune response, *C. trachomatis* has evolved to co-opt the IDO-driven cell-autonomous immune response as an inducer of bacterial quiescence. Accordingly, a humanized mouse model for chronic *C. trachomatis* infections would require both the removal of the murine IRG response and the recreation of IDO-mediated cell-autonomous immunity in urogenital epithelial cells.

## Materials and Methods

### Ethics statement

All experiments were approved by the Institutional Animal Care and Use Committee of Harvard Medical School. Harvard maintains an animal care and use program certified by The Association for the Assessment and Accreditation of Laboratory Animal Care (AAALAC) and all procedures are conducted in accordance with guidelines established by the American Veterinary Medical Association.

### Mice

All mice were maintained and bred under specific pathogen-free conditions. Control C57BL/6J (wildtype) B6.*Ifngr1* ^-/-^ and B6.*Ifng* ^-/-^ mice were obtained from The Jackson Laboratory. The targeted gene deletions of *Irgm1* and *Irgm3*, mice doubly deficient for *Irgm1* and *Irgm3* and NR1 mice expressing a TCR transgene specific for the *C. trachomatis* antigen Cta1 have been described previously [27], [28], [58], [59]. The NR1 T cell receptor transgene was crossed onto the *Irgm1* ^-/-^, *Irgm3* ^-/-^ and *Irgm1/m3* ^(-/-)^ genetic backgrounds.

### *C. trachomatis* strains and measurement of inclusion forming units and bacterial genomes

*C. trachomatis* serovar L2 434/Bu were propagated in McCoy cells and purified as described [17]. Cells were routinely cultured in high glucose DMEM (Gibco) supplemented with 10% fetal calf serum. For those experiments in which we monitored the antimicrobial effects of IDO-mediated tryptophan depletion, cells were transferred into DMEM F-12 culture medium lacking tryptophan (US Biological) supplemented with 3% fetal calf serum. Limiting the amount of calf serum was necessary to reduce the amount of serum-derived tryptophan to levels at which IFNγ-activated HeLa cells maximally restricted growth of *C. trachomatis*. Where indicated media was supplemented with tryptophan at a final concentration of 0.05 mg/ml. To quantify the bacterial load in *Chlamydia*-infected cells and in the uteri of infected animals, a previously described quantitative PCR assay was applied [17]. Briefly, total nucleic acid from infected cells or spleen homogenates was prepared using the QIAamp DNA mini kit from Qiagen. *Chlamydia* 16S DNA and mouse GAPDH DNA content of individual samples was then quantified by qPCR on an ABI 7000 sequence detection system using primer pairs and dual-labeled probes. Standard curves were generated from known amounts of *Chlamydia* and mouse DNA, and these curves were used to calculate the mass of *Chlamydia* DNA per unit mass of mouse DNA in the samples. Alternatively, infected cells in culture were lysed by sonication and combined with their culture supernatants. Serial dilutions of the lysate were applied to McCoy cell monolayers. Inclusions were counted by immunofluorescence microscopy 30 hours post-infection.

### Cell culture and *C. trachomatis* infections *ex vivo*, and microscopy

Mouse embryonic fibroblasts were generated from the indicated mouse strains as previously described [17]. Cells were treated with 100 U/ml recombinant mouse IFNγ (Chemicon International) over night before infection or left untreated. Cells were infected with *C. trachomatis* at a multiplicity of infection (MOI) of 2 in SPG buffer (220 mM sucrose, 12.5 mM phosphate, and 4 mM L-glutamic acid (pH 7.5) by centrifugation at 1928 x g for 1 h at 37°C and then returned to standard medium. *Chlamydia* inclusions were detected in infected cells using mouse anti-Hsc70 antibody (Abcam), followed by anti-mouse secondary fluorescently labeled antibody. Cell nuclei were visualized with 4′,6-diamidino-3-phenylindole (DAPI) staining and epifluorescent images were acquired with a Nikon Eclipse TE2000-U microscope using a Nikon Plan Apo 20x /0.75 N.A. Phase 2 objective. Images were saved as TIFF files and imported into Adobe Illustrator for labeling.

### Transfer of *C. trachomatis*-specific CD4^+^ T cells, anti-CD4 and anti-GR1 antibody depletion, and intrauterine infection

Before transfer, *C. trachomatis*-specific CD4^+^ T cells were isolated from peripheral lymphoid tissues of mice transgenic for the NR1 TCR (Vα2^+^, Vβ8.3^+^) and labeled with 5 µM carboxyfluorescein-diacetate-succinimidyl-ester (CFSE) in serum-free medium as described [26]. One day before an intrauterine infection with *C. trachomatis* recipient CD90.2^+^ mice were injected *i.v.* with 10^6^ lymphocytes derived from NR1 transgenic animals of the indicated genetic backgrounds and congenically marked with the surface marker CD90.1^+^. To infect the genital tract, mice were treated with 2.5 mg of medroxyprogestrone acetate s.c. and one week later 2*10^6^ inclusion-forming units of C. trachomatis L2 were instilled into the uterus using a commercially available non-surgical embryo transfer device (Paratechs). In order to deplete CD4^+^ T cells, mice were injected intraperitoneally with 0.5 mg anti-CD4^+^ mAb (GK 1.5) two days prior to infection and with 0.25 mg of the same antibody on days 0, 2, 5, 7, 9 and 12 post-infection. To deplete GR1^+^ cells, mice were injected with the anti-GR1^+^ depleting antibody RB6-8C5 and control mice were injected with the isogenic rat IgG2b antibody LTF-2 (BioXCell) following the same injection regiment.

### Tissue preparation and flow cytometry

At the indicated times post-infection, lymph nodes and uteri were collected. Uteri were digested with 1 mg/ml type XI collagenase and 50, 000 units/ml DNase for 45 minutes at 37°C. Single-cell suspensions were prepared for staining via mechanical disaggregation. Tissues were mechanically disaggregated and immediately stained for activation markers or stimulated for 5 h with 50 ng/ml PMA and 500 ng/ml ionomycin in the presence of brefeldin A to determine intracellular cytokine staining. Cells were preincubated with anti-FcRγ (Bio X-Cell) before staining with anti-CD4 Pacific Blue (Biolegend), anti-CD90.1 peridinin chlorophyll-a protein (BD Bioscience), and Live/Dead Aqua (Invitrogen). For activation marker analysis, we examined anti-CD62L allophycocyanin-Alexa 750 (Ebioscience), and anti-CD25 allophycocyanin (BD Bioscience). For intracellular staining, the following antibodies were used: anti-IFNγ-PE or -Alexa 700; anti-IL2-PE or -allophycocyanin; and anti-TNFα-PE or -PE-CyChrome 7 (BD Biosciences). Cells were permeabilized with the Cytofix/Cytoperm Plus kit according to the manufacturer's instructions (BD Bioscience). In all samples, an unbiased total of 10^6^ lymphocytes were collected based on forward and side scatter gating. Post-acquisition, lymphocytes were gated based on forward and side scatter, dead cells were excluded, and NR1^+^ cells were delineated by gating on CD4^+^, CD90.1^+^, Vα2^+^, Vβ8.3^+^ T cells events. Data were collected on a modified FACSCalibur (Cytek Development) or an LSRII (BD Bioscience) and analyzed using Flow Jo (Tree Star).

### Proliferation and cell death assay

Spleens were mechanically disaggregated, red blood cells were lysed and cell suspension was enriched for CD4^+^ T cells using the Dynal Mouse CD4^+^ Negative Isolation Kit (Invitrogen) according to the manufacturer's instructions. Isolated cells were CFSE labeled and 10^5^ cells were plated out in wells of a 96-well flat bottom plates coated with anti-CD3 (500 ng/ well) and anti-CD28 (50 ng/ well) antibodies. Three days later cell viability was assessed by flow cytometry with propidium iodide staining.

### T cell protection assay

CD4^+^ T cells were purified from NR1 mice using a mouse CD4^+^ isolation kit (Dynal; Invitrogen) per the manufacturer's directions. The T cells were cultured in RPMI 1640 (Invitrogen) supplemented with 10% FCS, L-glutamine, HEPES, 50 µM 2-ME, 50 U/ml penicillin, and 50 µg/ml streptomycin. To stimulate the T cells, irradiated feeder splenocytes were pulsed with 5 µM Cta1_133–152_ peptide and co-cultured with the CD4^+^-enriched NR1 cells at a stimulator to T cell ratio of 4∶1. To polarize T cells towards Th1, T cells were incubated with 10 ng/ml IL-12 (Peprotech) and 10 µg/ml anti-IL-4 (Biolegend) for 5–7 days. 10^7^ Th1-skewed *C. trachomatis*-specific CD4^+^ T cells were transferred into mice, and 24 h later mice were infected in the uterus as described above. Uteri were harvested 6 days after infection. To assess the protective capacity of the skewed cells, uteri from infected mice were homogenized, and DNA was prepared as described above and used for qPCR.

### Statistical analysis

All groups were evaluated for statistical significance through the use of unpaired two-tailed t tests. Where it appeared necessary to highlight significant differences between data points, the level of significance is depicted as: *, p<0.05; **, p<0.01; and ***, p<0.005.

## Supporting Information

Figure S1 **Induction of murine IDO1 by IFNγ is unable to restrict growth** ***C. trachomatis*** **.** (A) B6 MEFs were stimulated with IFNγ and the induction of IDO1, Irgb10 and Igtp was determined using qPCR. (B) B6 MEFs, *Irgm1/m3^(-/-)^* MEFs, and HeLa cells were stimulated with 100U IFNγ/ml (mouse or human, respectively) over night and subsequently infected with *C. trachomatis* in the presence or absence of tryptophan. Bacterial burden was determined using qPCR and compared to growth in unstimulated cells. (C) B6 MEFs transfected with empty vector or an expression vector for human IDO were infected with *C. trachomatis* in the presence or absence of tryptophan. Bacterial load was determined using qPCR. Significant differences between +/- trp (B) and control vector versus hIDO-expressing cells (C) were determined by two-tailed students t-test (*, p <0.05).
(0.10 MB TIF)

Figure S2 **Nos2 plays a minimal role in eliminating** ***C. trachomatis*** **in IRG-deficient mice.** Wildtype, *Irgm1/m3^(-/-)^Nos2^(-/-)^, Irgm1/m3^(-/-)^* and GR1-depleted *Irgm1/m3^(-/-)^* mice were transcervically infected with *C. trachomatis* and intrauterine bacterial burden was determined at 15 days post-infection. Intrauterine bacterial burden between the different mouse strains was not significantly different (n.s.). In contrast, GR1-depletion significantly increased bacterial burden in *Irgm1/m3^(-/-)^* mice (*, p<0.05).
(0.59 MB EPS)

Figure S3 **The ability of** ***Igrm1/m3^(-/-)^*** **mice to rapidly clear infections compared to** ***Ifngr1^(-/-)^*** **mice depends on the CD4+ and GR1+ cell compartments.** Wildtype, *Irgm1/m3^(-/-)^* and *Ifngr1^(-/-)^* mice were either treated with an isotype control antibody or with CD4- and GR1-depleting antibodies. At 15 days post-infection, intrauterine bacterial burden was determined by qPCR. Elevated burden in *Ifngr1^(-/-)^* mice compared to *Irgm1/m3^(-/-)^* mice was observed in the isotype control group (*, p<0.1), but not in the CD4/GR1-depleted group.
(0.47 MB EPS)
