# Supplementary figures and images for: Compensatory T Cell Responses in IRG-Deficient Mice Prevent Sustained Chlamydia trachomatis Infections
Source: PLoS Pathog. 2011 Jun 23;7(6):e1001346. doi: 10.1371/journal.ppat.1001346 (PMC3121881; doi:10.1371/journal.ppat.1001346)

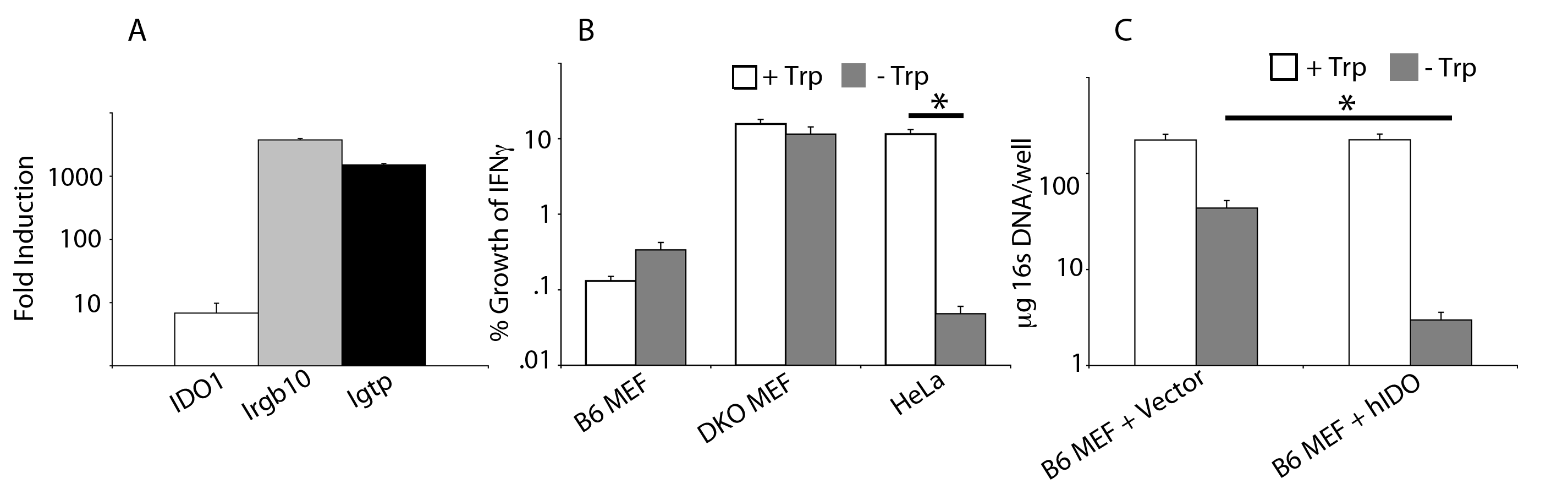

Supplement: Figure S1 — Induction of murine IDO1 by IFNγ is unable to restrict growth C. trachomatis . (A) B6 MEFs were stimulated with IFNγ and the induction of IDO1, Irgb10 and Igtp was determined using qPCR. (B) B6 MEFs, Irgm1/m3(-/-) MEFs, and HeLa cells were stimulated with 100U IFNγ/ml (mouse or human, respectively) over night and subsequently infected with C. trachomatis in the presence or absence of tryptophan. Bacterial burden was determined using qPCR and compared to growth in unstimulated cells. (C) B6 MEFs transfected with empty vector or an expression vector for human IDO were infected with C. trachomatis in the presence or absence of tryptophan. Bacterial load was determined using qPCR. Significant differences between +/- trp (B) and control vector versus hIDO-expressing cells (C) were determined by two-tailed students t-test (*, p <0.05). (0.10 MB TIF) [file ppat.1001346.s001.tif]
